# Dynamic changes of plasma extracellular vesicle long RNAs during perioperative period of colorectal cancer

**DOI:** 10.1080/21655979.2021.1943281

**Published:** 2021-07-16

**Authors:** Qing Hua, Wenhao Xu, Xuefang Shen, Xi Tian, Hailiang Zhang, Yan Li, Pingbo Xu

**Affiliations:** a Department of Anesthesiology, Fudan University Shanghai Cancer Center, Shanghai, China; b Department of Oncology, Shanghai Medical College, Fudan University, Shanghai, China; c Department of Anesthesiology, Zhongshan Hospital, Fudan University, Shanghai, China; d Department of Urology, Fudan University Shanghai Cancer Center, Shanghai, China; e Fudan University, Shanghai Cancer Center and Institutes of Biomedical Sciences, Fudan University, Shanghai, China

**Keywords:** Extracellular vesicles, long RNA sequencing, perioperative period, colorectal cancer

## Abstract

Extracellular vesicles (EVs) long RNAs (exLRs) have been shown to be indicators for the diagnosis and prognosis of colorectal cancer (CRC); however, the dynamic changes of exLRs during perioperative period and their cellular sources in CRC remains largely unknown. In this study, exLR sequencing (exLR-seq) was performed on plasma samples from three CRC patients at four time points (before surgery [T0], after extubation [T1], 1 day after surgery [T2], and 3 days after surgery [T3]). Bioinformatics approaches were used to investigate the profile and biofunctions of exLRs and their cellular sources. Greater than 12,000 mRNAs and 2,000 lncRNAs were reliably detected in each exLR-seq sample. Compared with T0, there were 110 differentially expressed genes (DEGs) in T1, 60 DEGs in T2, and 50 DEGs in T3. A total of 11 DEGs were found at all three time points and were related to membrane potential. In addition, compared to T0, 22 differentially expressed lncRNAs (DELRs) were found in T1, 19 DELRs in T2, and 38 DELRs in T3. Moreover, only three DELRs were detected at all three time points. Interestingly, EVs from CD8 + T cells, CD4+ memory T cells and NK cells decreased after surgery and the absolute quantity of EVs from immune cells were reduced as well. In summary, this study was the first to characterize the dynamic changes of exLRs during perioperative period and the cellular sources. These findings established the foundation for further studies involving the effects of these dynamically changed exLRs on CRC.

## Graphical Abstract

**Figure:**
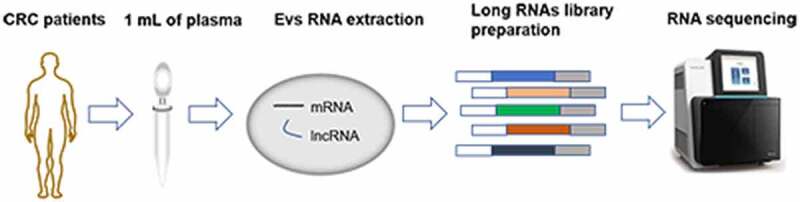

## Introduction

Colorectal cancer (CRC) is one of the most common malignancies worldwide [1,2]. According to the 2020 global cancer statistics, there are approximately 1.9 million newly diagnosed CRC patients and 935,000 CRC-related mortalities, accounting for 10% of global cancer cases and 9.4% of cancer-related deaths [3]. Despite the rapid development of advanced chemotherapy, targeted therapy and immunotherapy for tumors during the past decades, surgical resection remains the major treatment for CRC [4–7]. However, previous studies have demonstrated that surgical trauma causes long-term oncologic outcome by facilitating metastasis and recurrence of tumors [8,9]. During the perioperative period, a variety of factors participate in the metastasis and recurrence of primary tumors, such as dissemination of tumor cells, drugs used in anesthetic and analgesic procedures, destruction of the extracellular matrix, release of vascular endothelial growth factor (VEGF), post-operative immunosuppression [10]. Therefore, there is an urgent need to identify biomarkers involved in the postoperative metastasis and recurrence of tumors and evaluate the dynamic changes and biofunctions of them.

Extracellular vesicles (EVs) are lipid bilayer-enclosed, nanosized endocytic vesicles which could be secreted by most cell types [11,12]. EVs can modify the function of recipient cells by various bioactive contents, such as proteins (enzymes, extracellular matrix proteins, transcription factors, and receptors), DNAs, RNAs, and lipids [13]. It has been shown that EV long RNAs (exLRs), including circular RNA (circRNA), long non-coding RNA (lncRNA), and messenger RNA (mRNA), are abundant in human plasma [14–18]. ExLRs are considered to be valuable and functional [18,19] and play an important role in the progression of tumor development [20–22]. For example, Nabet *et al.* reported that an unshielded exosome RNA (RN7SL1) could act as a damage-associated molecular pattern (DAMP) to activate the pattern recognition receptor (PRR) RIG-I, driving anti-viral signaling when transferred to recipient breast cancer cells via an exosome, and ultimately leads to tumor growth and therapy resistance [16]. The CD274 mRNA in plasma-derived EVs is related to the response to anti-PD-1 antibodies in melanoma and non-small cell lung cancer [17]. In CRC, circulating EV microRNAs and lncRNAs are considered to be potential diagnostic biomarkers and related to mitomycin resistance [23–27]. These results showed that ExLRs could act as the cell-to-cell mediators of human cancers and promoted the progression of cancers. However, the dynamic changes of ExLRs during perioperative period and their biofunctions in the progression of CRC remains largely unknown.

In the current study, we first evaluated the expression profile of exLRs during the perioperative period. ExLRs sequencing (exLR-seq) was performed on plasma samples collected from three CRC patients at four specific timepoints (before surgery [T0], after extubation [T1], 1 day after surgery [T2], and 3 days after surgery [T3]) to detect the effects of surgical stress on the exLR expression profile. In addition, the biofunctions of the changed exLRs were also investigated to assess the effects of exLRs on CRC progression. Moreover, tracking the cellular source of circulating EVs provides biological information about the origin and the functional states [28]. However, the cellular origin of dynamic changes in circulating EVs during the post-operative period has not been thoroughly investigated. In this study, we also explored and compared the distinct cellular origins from plasma EV samples during the post-operative period.

Our study first evaluated the dynamic changes of exLRs during the perioperative period and their biological functions to find out appropriate biomarkers involved in the postoperative metastasis and recurrence of CRC. We also track the cellular sources of those dynamically changed exLRs to figure out the origin and functional states of these exLRs.

## Material and methods

### Patient specimens and clinical assessments

The present study recruited three CRC patients, all of whom underwent right hemicolectomy at Fudan University Shanghai Cancer Center by the same surgeon. All the participants were histologically confirmed to have colorectal adenocarcinoma (stage II) by two pathologists. Tumor staging was determined according to the AJCC Cancer Staging Manual. None of the patients received any other forms of therapy on the time of enrollment. This study was approved by the Ethics Committee of Fudan University Shanghai Cancer Center and informed written consent was obtained from all patients.

### Isolation of plasma from blood

Peripheral blood samples were collected from three CRC patients at four times (before surgery, after extubation, 1 day after surgery, and 3 days after surgery) in 10-mL EDTA-coated vacutainer tubes. Plasma was then separated by centrifugation at 3000 rpm (~800 × g) for 10 min at 25°C within 2 h after blood collection. Then, samples were centrifuged at 13,000 rpm (~16,000 × g) for 10 min at 4°C to remove debris. The plasma samples were then stored at – 80°C until use, according to a previous publication [18].

### Isolation of EVs and EV RNA

For every patient, 1 mL of plasma was used. EVs were isolated by affinity-based binding to spin columns via an exoRNeasy Serum/Plasma Kit (Qiagen, Hilden, Germany) according to the manufacturer’s instructions. Briefly, melted plasma was mixed with binding buffer and added to the exoEasy membrane affinity spin column. Samples were subjected to ultrafiltration using an Amicon Ultra-0.5 Centrifugal Filter 10 kDa (Merck Millipore, Germany) to reduce the eluate volume to 50 µL and exchange the buffer with phosphate buffer saline (PBS). For transmission electron microscopy (TEM), the size distribution measurement, and western blotting, the EVs were eluted with 400 μL of XE elution buffer, according to previous publications [29]. For TEM, ultrathin sections (100 nm) were cut using a LeicaUC6 ultra-microtome and post-stained with uranyl acetate for 10 min and with lead citrate for 5 min at room temperature before observation in a FEI Tecnai T20 TEM, operated at 120 kV. For EV RNA isolation, EVs were lysed on the column using QIAzol (Qiagen), and total RNA was then eluted and purified, as per other publications [30].

### ExLR-seq analysis

The strategy for exLR-seq analysis includes plasma preparation, isolation of EV and EV RNAs, RNA-seq library construction, sequencing, and data analysis. Briefly, to remove DNA, total EV RNA isolated from 1 mL of plasma was treated with DNase I (NEB; Ipswich, Massachusetts, USA). RNA-seq libraries were generated using SMART technology (Clontech). ExLR-seq was performed on an Illumina sequencing platform (San Diego, California, USA) with 150 bp paired-end run metrics. Gene expression levels were calculated in transcripts per kilobase

million (TPM). Annotations of mRNAs and lncRNAs were retrieved from the GENCODE database, according to previous publications [20,31].

### Identification of differentially expressed mRNAs and lncRNAs

Transcriptional profiles of EVs from the plasma of three CRC patients were evaluated during the perioperative period (before surgery [T0], after extubation [T1], 1 day after surgery [T2], and 3 days after surgery [T3]). Significantly differentially expressed genes (DEGs) and differentially-expressed lncRNAs (DELRs) were identified using the Limma R package (version 3.6.3) with a |logFC| > 1 and p < 0.1.

### Functional enrichment analysis of DEGs and DELRs

The intersective hub genes during the perioperative period were selected for further analyses using a Venn diagram. A protein–protein interaction (PPI) network of hub genes was constructed using GeneMANIA (http://genemania.org/). Biological processes, cellular components, and molecular function of gene ontology (GO) functional analysis and Kyoto encyclopedia genes and genomes (KEGG) pathway were predicted using the Web-based Gene seT AnaLysis Toolkit (WebGestalt [http://www.webgestalt.org/]) and visualized using R software. In the functional analyses, the input parameters including gene names of all the DEGs, gene ontology (GO) and Kyoto encyclopedia genes and genomes (KEGG) pathways and the results can change depending on the query/input information.

### Western blot analysis

Fifty milligrams of exosomes were extracted using 2X SDS lysis buffer, separated by 4%–12% SDS-PAGE, transferred to a PVDF membrane, blocked with 5% BSA in TBST, and probed with specific primary antibodies against Calnexin (1:1000 dilution; Abcam, Cambridge, UK), CD63 (1:1000 dilution; Abcam), and TSG101 (1:1500 dilution; Abcam). β-actin (1:5000 dilution; Santa Cruz Biotechnology, Inc., Santa Cruz, CA, USA) was used as a loading control. The chemiluminescent signals were detected with a chemiluminescence imaging system and quantified by Image J software (v1.37).

### Data and statistical analyses

All statistical analyses were two-sided. A |logFC| > 1 and p < 0.05 were considered statistically significant. The following R software packages were used in this study: e1071, glmnet, varSelRF, pROC, and caret. Nonparametric T test was used in Limma Package and the comparison of immune cells producing EVs before and after surgery. Hypergeometric test was used in the functional enrichment analysis. Spearman’s rank correlation coefficient was utilized in the correlation analysis of different types of immune cells.

## Results

Firstly, we evaluated the dynamic changes of mRNAs and lncRNAs during the perioperative period via ExLR-seq. Then, we detected the biological functions of these DEGs to find out appropriate biomarkers involved in the postoperative metastasis and recurrence of CRC. Finally, we track the cellular sources of those dynamically changed exLRs to figure out the origin and functional states of these exLRs.

### Isolation and identification of EVs

**Figure 1.**
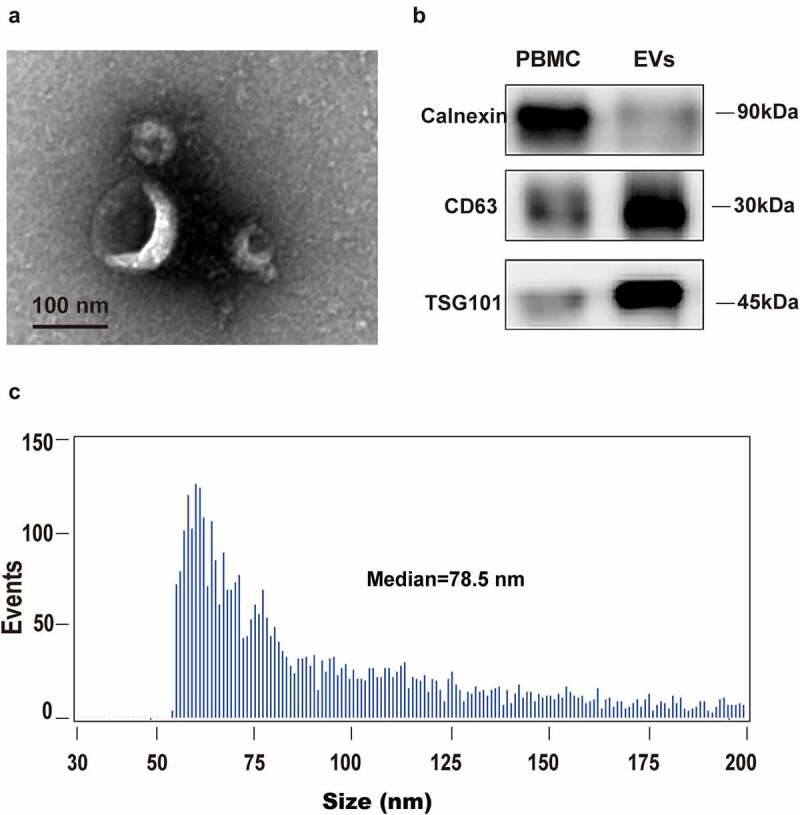
**Human blood EVs confirmation**. EVs were isolated and purified from plasma using membrane affinity spin columns. (a) Electron microscopy image of isolated vesicles. (b) Size distribution measurements of isolated vesicles. (c) Western blots of calnexin, which can be detected in PBMCs, but not in isolated vesicles, was used as a control. EV markers TSG101 and CD63 in isolated vesicles were detected in EVs, but not in PBMCs

To evaluate the integrity of isolated EVs from plasma, EV morphology was inspected by electron microscopy. As shown in Figure 1, the isolated vesicles in plasma were cup-shaped, rounded, and double membrane-bound vesicle-like (Figure 1a). Furthermore, flow cytometry exhibited a heterogeneous population of spherical nanoparticles, with abundant peaks ranging from 50 to 200 nm (Figure 1b). In addition, western blot analysis revealed characteristic exosomal marker (CD63 and TSG101) expression in isolated vesicles, but not in peripheral blood mononuclear cells (PBMCs). Calnexin, which is an intracellularly enriched protein in PBMCs and often used as a negative-control protein marker for EV identification, was detected in PBMCs, but not in isolated vesicles (Figure 1c). These data indicated that the isolated vesicles were composed mostly of exosomes.

### Dynamic changes of mRNAs in EVs before and after surgery

**Figure 2.**
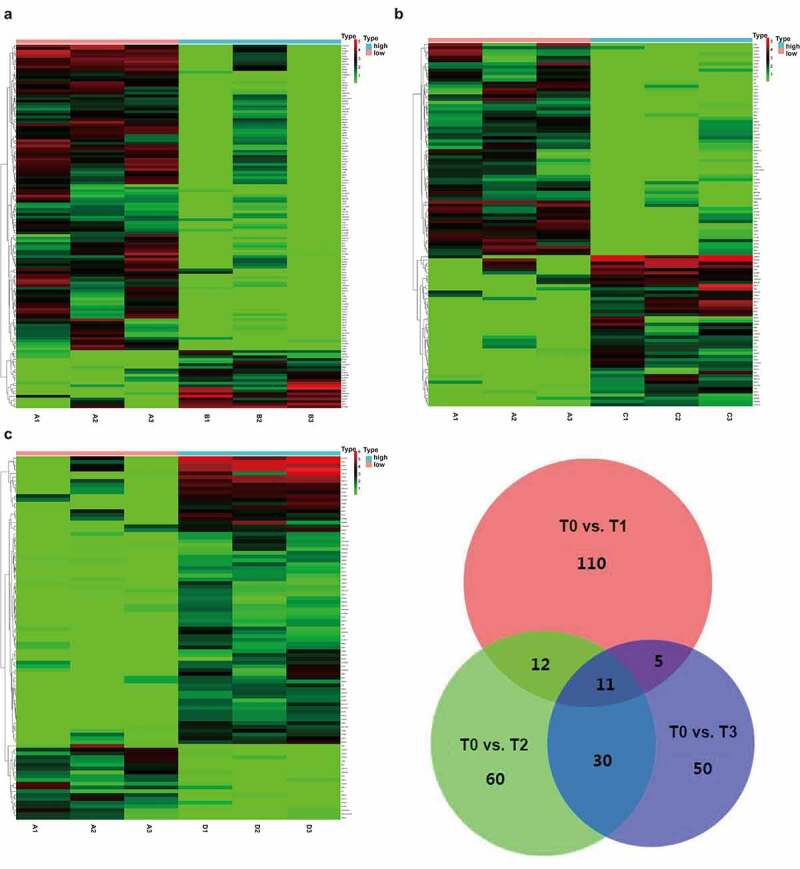
**Comprehensive mRNAs in extracellular vesicles and functional annotations before and after surgery**. (a-c) Significant DEGs in extracellular vesicles were screened and identified using the ‘Limma’ R package between samples before surgery and after extubation (Fig. A), or 1 day after surgery (Fig. B), or 3 days after surgery (Fig. C). (d) A total of 11 common DEGs were obtained in extracellular vesicles before and different time points after surgery using a Venn diagram

ExLR-seq was conducted using plasma samples from three CRC patients at four timepoints. Approximately 12,924 mRNAs were reliably detected in each sample. Dynamic changes were observed in the expression profiles of mRNAs in EVs during the postoperative period. Briefly, as shown in Figures 2A, 110 DEGs in T1 were compared with those in T0. In addition, compared with T0, there were 60 DEGs in T2 (Figure 2b) and 50 DEGs in T3 (Figure 2c). Taking the intersection of DEGs in the three groups revealed that a total of 11 DEGs (hub DEGs), including *DGKI, GRB14, KIAA1549, WT1, ACKR4, PLXNB3, KCNH8, TCTEX1D1, ILDR2, DYTN* and *CHRNA2*, changed at all three timepoints compared to T0 (Figure 2d). Although it did not reach significance owing to the sample size, there existed an obvious trend (logFC| > 1 and p < 0.1) and details are shown in table 1 (Table 1, Fig S1).

### Functional annotations of mRNAs in EVs

**Figure 3.**
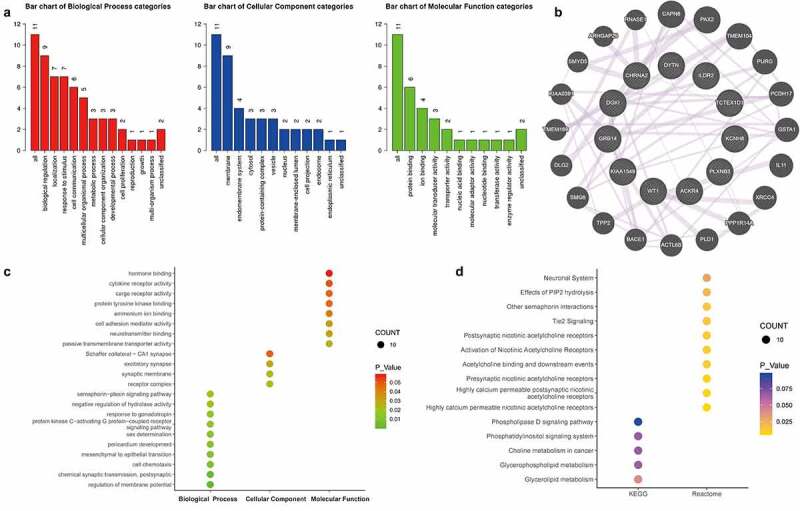
**The functional annotations of mRNAs in EVs**. (a) Biological processes, cellular components, and molecular function analysis from GO items of 11 hub genes were evaluated. (b) The protein–protein interaction network was used, showing direct interactions and potential associations between proteins. (c) The 11 hub genes most significantly involved in changed GO functions. (d) Significantly altered KEGG and Reactome pathways were predicted

Next, functional annotations of 11 altered mRNAs in EVs were determined. With respect to the biological process, nine of the 11 DEGs were involved in the biological regulation process and seven DEGs participated in the localization process and stimulus response. For the cellular component, nine DEGs were membrane components. In addition, molecular function analysis showed that six DEGs were protein-binding mRNAs (Figure 3a). To further determine the interactions among the 11 DEGs, the protein–protein interaction network was used (Figure 3b). Moreover, GO function analysis showed that the 11 hub DEGs most significantly involved in the regulation of membrane potential, cell chemotaxis, chemical synaptic transmission, and mesenchymal-epithelial transition (Figure 3c). KEGG pathway analysis revealed that the hub DEGs were enriched for some pathways, such as nicotinic acetylcholine receptors activities, Tie2 signaling, other semaphoring interactions, and choline and glycerolipid metabolism (Figure 3d).

### Dynamic changes of lncRNAs in EVs before and after surgery

**Figure 4.**
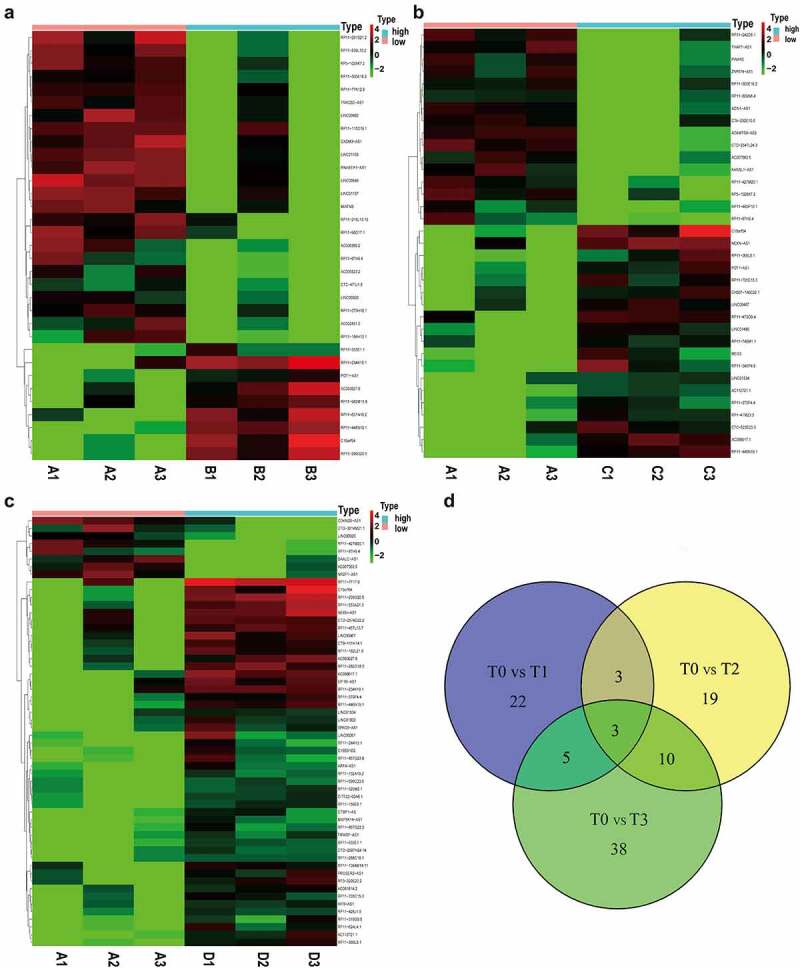
**Comprehensive lncRNAs in EVs before and after surgery**. (a-c) Significant lncRNAs in EVs were also screened and identified using the ‘Limma’ R package between samples before surgery and after extubation (Fig. A), or 1 day after surgery (Fig. B), or 3 days after surgery (Fig. C). (d) A total of 3 common DEGs were obtained in EVs before and different time points after surgery using a Venn diagram

Except for mRNAs, approximately 2200 lncRNAs were detected in each sample. Dynamic changes were also observed in the expression profiles of lncRNAs in EVs during the post-operative period. As shown in Figure 4a, we identified 22 DELRs between T0 and T1. In addition, 19 DELRs were detected between T0 and T2 (Figure 4b) and 38 DELRs between T0 and T3 (Figure 4c). Furthermore, we found that only three DELRs (*C15orf54, RP11-446N19.1*, and *RP11-87H9.4*) changed at all three timepoints (Figure 4d). Although it did not reach significance owing to the sample size, there existed an obvious trend (logFC| > 1 and p < 0.1) and details are shown in table 2 (Table 2, Fig S2).

### Cell source analysis of EVs

**Figure 5.**
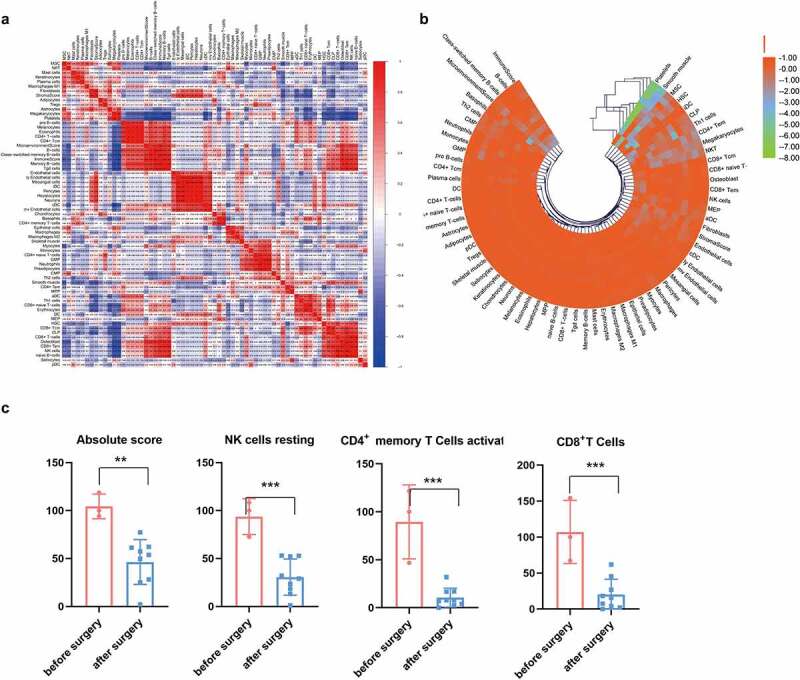
**Cell source analysis of EVs**. (a) The correlation of immune and stroma cell expression derived from EVs. (b) Dynamic changes of cells derived from EVs before and after surgery. (c) Comparison of immune cells producing EVs before and after surgery. * p < 0.05, ** p < 0.01, *** p < 0.01 vs before surgery

Because blood EVs are derived from a variety of tissues, the xCell tool (http://xcell.ucsf.edu) was used to characterize the proportions of cell types derived from EVs. xCell is a webtool that performs cell-type enrichment analysis from gene expression data for 64 immune and stroma cell types. xCell is a gene signature-based method learned from thousands of pure cell types from various sources. xCell applies a novel technique for reducing associations between closely related cell types. We identified 67 immune and stroma cell types and evaluated the correlation between immune and stroma cells (Figure 5a). Dynamic changes of the origination of EVs during the postoperative period were also investigated (Figure 5b). Consensus clustering were utilized to explore potential clusters and Consensus clustering (or aggregated clustering) is a robust approach that relies on multiple iterations of the chosen clustering method on sub-samples of the dataset. Specifically, EVs derived from platelets were gradually reduced after surgery. Clinically, the immunosuppressive microenvironment caused by surgery can lead to tumor metastases and recurrence and the role of EVs in immune regulation has been intensively studied. Following infection, the release of EVs carrying immunomodulatory molecules by various immune cells can influence primary and secondary immune responses [32,33]. Therefore, we further investigated the changes in immune cells derived from EVs before and after surgery. As shown in Figure 5c, the total number of EVs derived from immune cells decreased after surgery. EVs derived from CD8 + T, CD4+ memory T, and natural killer (NK) cells decreased as well (Figure 5c).

## Discussion

Recently, EVs, as a molecular approach to analyze tumor diagnosis and progression, has attracted more and more attention [31,34]. While studies of EVs on cancer development have expanded rapidly, few studies have investigated the influence of surgical stress on exLRs and the cellular origins of exLRs. Our research showed that there are a large variety of exLRs in human blood. Given the high abundance and heterogeneity of blood exLRs, we intended to find the differences of expression profile and function in exLRs before and after surgery and their influence on CRC progression and prognosis.

As the size similarity between exosomes and other EVs, including ectosomes and MVs, has deeply hindered the development of isolation processes. It is critical to pay attention to the isolation of plasma, EV purification and preparation of EV RNA in order to obtain reliable exLR-seq data. In this study EVs were isolated via density gradient centrifugation (DG), which was intensively used in plasma and cell culture supernatants. Although the process is time-consuming and highly instrument-dependent, DG is easy to perform and yields exosomes with higher purity and size uniformity. The proportion of exosomes in the isolated EVs was confirmed from the aspect of morphology, particle size, and characteristic exosomal markers (CD63 and TSG101). Rapid development of EV isolation technology makes it possible to use EVs and exLRs for further cancer-related studies.

In this study, surgical stress caused significant changes to the expression profile of exLRs. 11 mRNAs, including *DGKI, GRB14, KIAA1549, WT1, ACKR4, PLXNB3, KCNH8, TCTEX1D1, ILDR2, DYTN* and *CHRNA2*, have dynamically changed at all three timepoints compared with T0. Several of these exLRs were associated with CRC progression. For example, WT1 expression in CRC primary tumors could be a novel independent marker for prognosis and tumor progression [35,36]. GRB14, which belongs to a small family of adapter proteins, could encode a growth factor receptor-binding protein that interacts with insulin receptors and insulin-like growth factor receptors (IGF-Rs) [37]. IGF-1 R expression is associated with tumor progression and poor prognosis in several cancer types, including gastrointestinal malignancies [38]. *KIAA1549* belongs to the UPF0606 family and is related to oncogenic MAPK signaling [39]. ACKR4, which is a receptor for C–C type chemokines, has been shown to bind T cells and dendritic cell-activated chemokines and plays a significant role in controlling the migration of immune and cancer cells [40]. These findings indicated that mRNAs dynamically changed during the peri-operative period may play a role in the development of CRC. However, the exact effect of these mRNAs and the underlying mechanisms require further investigation.

Our data also showed that lncRNAs are enriched in EVs; however, only three common lncRNAs were detected at three time points compared with T0. This finding may due to our small sample size. In a corollary study, we will expand our samples to further explore the exact effect of surgery on EVs and the underlying mechanism.

Innate immune cells, such as NK cells, neutrophils, mast cells (MCs), macrophages, eosinophils and adaptive immune cells, including DCs, T cells, and B cells, derived from exosomes can directly interact with cancer cells and uptake by tumor cells inducing different types of immune responses [41,42]. Accumulating evidence has shown that EVs derived from immune cells can promote pro-tumor and anti-tumor immunity, which suggests a complex relationship between the immune cell-derived EVs and the immune system [43–46]. NK cells that were previously exposed to neuroblastoma (NB) can secrete exosomes containing NK cell receptors, such as CD56, KIR2DL2, and NKG2D receptors, which can subsequently stimulate normal NK cells, generating greater and more efficient cytotoxicity against NB tumor cells [45]. CD8 + T cell-derived exosomes with membrane expression of Fas ligand (FasL) can promote invasion and metastasis of Fas+ tumor cells through matrix metalloproteinase-9 (MMP-9)-mediated degradation of extracellular matrix proteins [43]. In our study, we found that total EVs derived from immune cells, especially from CD8 + T cells, CD4+ memory T cells and NK cells, decreased after surgery. Further studies are warranted to fully characterize EVs derived from immune cells, and to learn how to precisely engineer exosomes for therapeutic antitumor treatment.

**Table:** Significant DELRs between T0 and T1

LncRNA	Mean(T0)	Mean(T*1*)	logFC	pValue	FDR
AC002451.3	2.21713388	0.114178067	−4.27934	0.0765225	0.683824
AC005523.2	1.52729331	0.0228,35613	−6.06355	0.0765225	0.683824
AC006369.2	3.413328513	0.091342467	−5.22375	0.0765225	0.683824
AC093627.8	0.325408993	6.553166567	4.331866	0.0765225	0.683824
C15orf54	0.081352247	11.77879127	7.177794	0.0765225	0.683824
CADM3-AS1	6.056916993	0.548054667	−3.46619	0.0765225	0.683824
CTC-471J1.8	0.74328401	0.091342467	−3.02456	0.0765225	0.683824
LINC00649	8.314309343	0.548054667	−3.9232	0.0765225	0.683824
LINC00662	5.140611187	0.411041	−3.64459	0.0765225	0.683824
LINC00920	1.453565963	0.1370137	−3.4072	0.0765225	0.683824
LINC01133	5.082520677	0.479548	−3.4058	0.0765225	0.683824
LINC01137	5.426904483	0.662232667	−3.03472	0.0765225	0.683824
MIATNB	4.27227625	0.433876667	−3.29965	0.0765225	0.683824
POT1-AS1	0.10169031	1.446493733	3.830306	0.0765225	0.683824
RNASEH1-AS1	5.546831097	0.502383667	−3.4648	0.0765225	0.683824
RP11-115D19.1	4.29410445	1.141780667	−1.91107	0.0765225	0.683824
RP11-164H13.1	2.22593248	0.0228,35613	−6.60698	0.0765225	0.683824
RP11-216 L13.19	3.491072927	0.416797333	−3.06625	0.0765225	0.683824
RP11-234K19.1	0.750527333	11.01633047	3.875595	0.0765225	0.683824
RP11-291B21.2	7.87761297	0.1370137	−5.84537	0.0765225	0.683824
RP11-299G20.5	0.081352247	6.911403467	6.408653	0.0765225	0.683824
RP11-303E16.2	2.226079677	0.1826849	−3.60708	0.0765225	0.683824
RP11-333E1.1	0.041695967	1.0917443	4.710583	0.0765225	0.683824
RP11-379H18.1	2.29573854	0.342534333	−2.74464	0.0765225	0.683824
RP11-446N19.1	0.062543933	5.9012772	6.560013	0.0765225	0.683824
RP11-539L10.2	4.57341401	0.1370137	−5.06088	0.0765225	0.683824
RP11-631N16.2	0.245482033	6.750055167	4.78121	0.0765225	0.683824
RP11-77K12.9	2.799639297	0.525219	−2.41425	0.0765225	0.683824
RP11-87H9.4	2.333000947	0.0228,35613	−6.67476	0.0765225	0.683824
RP11-95D17.1	4.896657887	0.269692367	−4.18241	0.0765225	0.683824
RP11-982M15.8	0.508451553	2.996266867	2.558984	0.0765225	0.683824
RP5-1028K7.2	3.882608593	0.274027367	−3.82463	0.0765225	0.683824
TNRC6C-AS1	2.94829249	0.411041	−2.84253	0.0765225	0.683824

**Table:** Significant DELRs between T0 and T2

LncRNA	Mean(T0)	Mean(T2)	logFC	pValue	FDR
AC007563.5	2.76586097	0.155973413	−4.14836	0.0765225	0.621384
AC098617.1	0.166783833	4.994414183	4.904264	0.0765225	0.621384
AC112721.1	0.02084798	0.913731333	5.453791	0.0765225	0.621384
ADAMTS9-AS2	4.195305597	0.038,993,353	−6.7494	0.0765225	0.621384
AZIN1-AS1	3.07585092	0.17547009	−4.13169	0.0765225	0.621384
C15orf54	0.081352247	23.33072509	8.163833	0.0765225	0.621384
CH507-145 C22.	0.38642318	2.695785687	2.802452	0.0765225	0.621384
CTA-292E10.6	3.03295283	0.584900297	−2.37446	0.0765225	0.621384
CTC-523E23.3	0.542047333	3.586782283	2.726199	0.0765225	0.621384
CTD-2547L24.3	5.05390719	0.038993353	−7.01803	0.0765225	0.621384
KANSL1-AS1	3.431506167	0.05849003	−5.87451	0.0765225	0.621384
LINC00467	0.406761243	3.144939917	2.950778	0.0765225	0.621384
LINC01480	0.1022842	2.300809347	4.491486	0.0765225	0.621384
LINC01534	0.271023733	1.089082163	2.006622	0.0765225	0.621384
MEG3	0.020456837	2.239088517	6.774185	0.0765225	0.621384
NEXN-AS1	0.833860547	9.482922053	3.507454	0.0765225	0.621384
POT1-AS1	0.10169031	2.667381173	4.71317	0.0765225	0.621384
PWAR5	3.006275807	0.136476737	−4.46125	0.0765225	0.621384
RP11-242D8.1	4.363404197	0.409430207	−3.41376	0.0765225	0.621384
RP11-303E16.2	2.226079677	0.38993353	−2.51321	0.0765225	0.621384
RP11-345P4.9	0.0613705	4.858066433	6.306693	0.0765225	0.621384
RP11-366 L5.1	0.020338063	3.031805823	7.219851	0.0765225	0.621384
RP11-379F4.4	0.125087867	1.32542017	3.405436	0.0765225	0.621384
RP11-427M20.1	3.052397903	0.102365933	−4.89814	0.0765225	0.621384
RP11-446N19.1	0.062543933	3.929752793	5.973425	0.0765225	0.621384
RP11-479O9.4	0.777359667	4.55295743	2.55015	0.0765225	0.621384
RP11-495P10.1	1.398293977	0.077986707	−4.1643	0.0765225	0.621384
RP11-705 C15.3	0.162704497	2.89037666	4.150931	0.0765225	0.621384
RP11-746M1.1	0.3273094	1.68878291	2.367257	0.0765225	0.621384
RP11-809N8.4	1.43980743	0.253456793	−2.50606	0.0765225	0.621384
RP11-87H9.4	2.333000947	0.020473183	−6.83231	0.0765225	0.621384
RP1-47M23.3	0.041695967	1.749010003	5.390487	0.0765225	0.621384
RP5-1028K7.2	3.882608593	0.368517333	−3.39722	0.0765225	0.621384
THAP7-AS1	4.052557717	0.136476737	−4.89211	0.0765225	0.621384
ZNF674-AS1	3.01449014	0.214463443	−3.81311	0.0765225	0.621384

**Table:** 

LncRNA	Mean(T0)	Mean(T3)	logFC	pValue	FDR
AC007563.5	2.76586097	0.215818967	−3.67984	0.0765225	0.355949
AC091814.2	0.223718683	1.485052283	2.730756	0.0765225	0.355949
AC093627.8	0.325408993	4.232574173	3.701209	0.0765225	0.355949
AC098617.1	0.166783833	5.820427687	5.125074	0.0765225	0.355949
AC112721.1	0.02084798	1.709872217	6.357837	0.0765225	0.355949
ARF4-AS1	0.040913667	0.893591417	4.448961	0.0765225	0.355949
BAALC-AS1	2.832459073	0.172655167	−4.03609	0.0765225	0.355949
C15orf54	0.081352247	12.67610939	7.283714	0.0765225	0.355949
CDKN2B-AS1	4.44231614	0.36374714	−3.6103	0.0765225	0.355949
CITF22-92A6.1	0.081827333	0.85146136	3.379286	0.0765225	0.355949
CTB-111H14.1	0.28473287	2.847558803	3.322045	0.0765225	0.355949
CTBP1-AS	0.041695967	0.94928833	4.508867	0.0765225	0.355949
CTD-2574D22.2	0.833860547	3.866681563	2.213218	0.0765225	0.355949
CTD-2587H24.1	0.1042399	0.609993183	2.548886	0.0765225	0.355949
CTD-3014M21.1	3.569017227	0.161665397	−4.46444	0.0765225	0.355949
CYB561D2	0.040676123	1.130932207	4.797186	0.0765225	0.355949
EIF1B-AS1	0.542047333	4.039256577	2.897599	0.0765225	0.355949
KIF9-AS1	0.18304256	0.99462356	2.441971	0.0765225	0.355949
LINC00261	0.040913667	2.71680064	6.053182	0.0765225	0.355949
LINC00467	0.406761243	4.464962843	3.456394	0.0765225	0.355949
LINC00920	1.453565963	0.080832697	−4.16851	0.0765225	0.355949
LINC01002	0.166783833	1.508721053	3.177275	0.0765225	0.355949
LINC01534	0.271023733	1.066834697	1.976846	0.0765225	0.355949
MAP3K14-AS1	0.02084798	0.650734907	4.96409	0.0765225	0.355949
NEXN-AS1	0.833860547	8.247496673	3.306078	0.0765225	0.355949
NR2F1-AS1	3.88181485	0.172655167	−4.49077	0.0765225	0.355949
PROSER2-AS1	0.2250252	1.895535413	3.074447	0.0765225	0.355949
RP11-1094M14.	0.3273094	1.935723837	2.564146	0.0765225	0.355949
RP11-156E8.1	0.081827333	0.919915587	3.490847	0.0765225	0.355949
RP11-182 L21.6	0.223718683	3.090707643	3.78818	0.0765225	0.355949
RP11-234K19.1	0.750527333	4.799530327	2.676917	0.0765225	0.355949
RP11-244H3.1	0.020456837	0.80383261	5.29624	0.0765225	0.355949
RP11-282O18.3	0.162704497	4.722175677	4.859126	0.0765225	0.355949
RP11-288C18.1	0.1042399	0.553462837	2.408579	0.0765225	0.355949
RP11-299G20.5	0.081352247	9.772892627	6.90846	0.0765225	0.355949
RP11-319G9.5	0.020338063	0.97249277	5.579433	0.0765225	0.355949
RP11-320 M2.1	0.1022842	0.71546583	2.8063	0.0765225	0.355949
RP11-333E1.1	0.041695967	0.642504777	3.945727	0.0765225	0.355949
RP11-366 L5.1	0.020338063	1.697460663	6.383052	0.0765225	0.355949
RP11-379F4.4	0.125087867	2.12802338	4.0885	0.0765225	0.355949
RP11-427M20.1	3.052397903	0.028775863	−6.72894	0.0765225	0.355949
RP11-428J1.5	0.10169031	0.803844833	2.982735	0.0765225	0.355949
RP11-446N19.1	0.062543933	2.991808677	5.580004	0.0765225	0.355949
RP11-467L13.7	0.650817987	3.83507436	2.558929	0.0765225	0.355949
RP11-553A21.3	0.18304256	6.535466787	5.158039	0.0765225	0.355949
RP11-596 C23.6	0.1022842	0.54519963	2.414201	0.0765225	0.355949
RP11-624L4.1	0.020338063	1.489609253	6.194608	0.0765225	0.355949
RP11-705 C15.3	0.162704497	1.189071987	2.86951	0.0765225	0.355949
RP11-732A19.2	0.081827333	0.596647493	2.866224	0.0765225	0.355949
RP11-7F17.8	1.403326287	18.64460099	3.731836	0.0765225	0.355949
RP11-867G23.3	0.041695967	0.716191363	4.102365	0.0765225	0.355949
RP11-867G23.8	0.020338063	1.565923	6.266687	0.0765225	0.355949
RP11-87H9.4	2.333000947	0.028775863	−6.34118	0.0765225	0.355949
RP3-329E20.2	0.204568367	2.282784317	3.48014	0.0765225	0.355949
SPAG5-AS1	0.145935867	2.191379437	3.908433	0.0765225	0.355949
TIPARP-AS1	0.083391933	0.705930717	3.081547	0.0765225	0.355949

**Table:** Significant DEGs between T0 and T1

GENE	Mean(T0)	Mean(T1)	logFC	pValue	FDR
TSPYL5	8.297698333	1	−3.05271	0.0636026	0.381797
NAGPA	8.164393	1	−3.02935	0.0636026	0.381797
ZNF112	7.031611667	1	−2.81386	0.0636026	0.381797
GSTZ1	7.736256333	1	−2.95164	0.0636026	0.381797
SLC25A33	4.360061333	1	−2.12435	0.0636026	0.381797
C1orf186	5.760208	1	−2.52612	0.0636026	0.381797
CC2D2A	5.340496	1	−2.41697	0.0636026	0.381797
DCLK1	7.265278667	1	−2.86102	0.0636026	0.381797
CXorf57	4.585493667	1	−2.19708	0.0636026	0.381797
IL2RA	3.627303333	1	−1.8589	0.0636026	0.381797
C2orf81	6.706935333	1	−2.74565	0.0636026	0.381797
RPUSD2	8.42355	1	−3.07443	0.0636026	0.381797
RABL2A	5.830274	1	−2.54356	0.0636026	0.381797
AC092835.	6.381238667	1	−2.67384	0.0636026	0.381797
WT1	5.004191667	1	−2.32314	0.0636026	0.381797
GCNT4	4.203905	1	−2.07173	0.0636026	0.381797
TIGD2	5.404809	1	−2.43424	0.0636026	0.381797
MYT1	2.737559667	1	−1.45289	0.0636026	0.381797
C14orf80	4.038967333	1	−2.01399	0.0636026	0.381797
GOLGA7B	5.057243333	1	−2.33835	0.0636026	0.381797
UNC13 C	3.131791333	1	−1.64699	0.0636026	0.381797
ZNF705A	5.314469	1	−2.40993	0.0636026	0.381797
POC1A	3.826612667	1	−1.93607	0.0636026	0.381797
TIFAB	7.848899333	1	−2.97249	0.0636026	0.381797
NLRP7	5.482675333	1	−2.45488	0.0636026	0.381797
CFAP53	5.940012	1	−2.57047	0.0636026	0.381797
TCTEX1D1	1	4.620729	2.20812	0.0636026	0.381797
VNN3	1	10.84045167	3.438353	0.0636026	0.381797
HTR2A	1	3.366183667	1.751114	0.0636026	0.381797
ILDR2	1	5.065631333	2.340742	0.0636026	0.381797
EP400NL	15.092232	2.712671	−2.47602	0.0765225	0.381797
DCPS	14.96914533	2.027602667	−2.88414	0.0765225	0.381797
ZNF354 C	11.624222	1.205520667	−3.26941	0.0765225	0.381797
FLT4	8.531011667	1.616561667	−2.39979	0.0765225	0.381797
PTPRO	13.12034167	2.027602667	−2.69396	0.0765225	0.381797
RITA1	7.793430333	1.159849333	−2.74832	0.0765225	0.381797
KCNH8	6.572459333	1.159849333	−2.5025	0.0765225	0.381797
CCDC167	15.463013	2.210287667	−2.80652	0.0765225	0.381797
TRPV2	10.67998933	1.959095667	−2.44665	0.0765225	0.381797
A4GALT	7.112375333	1.753575333	−2.02003	0.0765225	0.381797
GALK1	10.69892733	2.895356	−1.88565	0.0765225	0.381797
LTBP2	9.837398333	1.662232667	−2.56515	0.0765225	0.381797
TMEM234	6.152497	1.274027333	−2.27178	0.0765225	0.381797
DNALI1	7.875622	1.114178	−2.82141	0.0765225	0.381797
NEK3	10.27452633	1.760042	−2.54539	0.0765225	0.381797
MTFP1	9.792329333	1.433876667	−2.77173	0.0765225	0.381797
SELO	8.185779	1.114178	−2.87714	0.0765225	0.381797
PCED1A	9.752530333	1.959095667	−2.31559	0.0765225	0.381797
GSTCD	10.69255167	1.159849333	−3.2046	0.0765225	0.381797
MMP23B	5.119569333	1.479548	−1.79087	0.0765225	0.381797
FOXRED1	7.648389	1.639397333	−2.22199	0.0765225	0.381797
C9orf172	7.559422667	2.210287667	−1.77404	0.0765225	0.381797
FAM206A	12.360496	1.913424667	−2.69151	0.0765225	0.381797
MIPEP	12.83586	2.118945	−2.59876	0.0765225	0.381797
NUDT16L1	10.97319067	2.210287667	−2.31168	0.0765225	0.381797
PEMT	8.485709667	1.890589	−2.1662	0.0765225	0.381797
GPSM1	4.377577333	1.122587333	−1.9633	0.0765225	0.381797

**Table:** 

CDCA5	9.481528667	1.114178	−3.08914	0.0765225	0.381797
TRIM66	9.392559333	1.799246333	−2.38413	0.0765225	0.381797
EVC	7.847389333	1.479548	−2.40706	0.0765225	0.381797
OSBPL7	7.09137	1.228356	−2.52934	0.0765225	0.381797
IL23A	6.11104	1.388205333	−2.1382	0.0765225	0.381797
GYPE	12.21936	2.552821667	−2.259	0.0765225	0.381797
ATG9B	6.718192	1.269692333	−2.40359	0.0765225	0.381797
CHTF18	4.928835333	1.205520667	−2.03159	0.0765225	0.381797
NDOR1	5.756575333	1.593726	−1.85281	0.0765225	0.381797
TMEM150A	6.262062	1.182685	−2.40457	0.0765225	0.381797
LRFN3	4.199165	1.525219	−1.46109	0.0765225	0.381797
WDR83	9.817685333	2.804013667	−1.80789	0.0765225	0.381797
HMCN1	5.950431667	1.433876667	−2.05307	0.0765225	0.381797
ASB13	9.439516	1.411041	−2.74195	0.0765225	0.381797
BCDIN3D	5.399809	1.220657333	−2.14525	0.0765225	0.381797
S1PR2	5.867014667	1.045671333	−2.4882	0.0765225	0.381797
AMDHD1	5.082083667	1.525219	−1.7364	0.0765225	0.381797
SLC25A17	7.005707	1.593726	−2.13613	0.0765225	0.381797
ALG1	4.579064	1.045671333	−2.13062	0.0765225	0.381797
SSPN	6.093317667	2.152322	−1.50133	0.0765225	0.381797
POGLUT1	6.763843667	1.662232667	−2.02472	0.0765225	0.381797
PARS2	4.567606333	1.0228,35667	−2.15886	0.0765225	0.381797
STK32B	5.824938333	1.570890333	−1.89066	0.0765225	0.381797
FAM207A	8.498970333	1.616561667	−2.39436	0.0765225	0.381797
LRWD1	9.251122333	1.365369667	−2.76034	0.0765225	0.381797
ERP27	4.538457667	1.319698667	−1.78199	0.0765225	0.381797
UBE3D	6.706291	1.685068333	−1.99271	0.0765225	0.381797
RAG1	6.426811	1.342534333	−2.25914	0.0765225	0.381797
THNSL1	6.241041333	1.411041	−2.14503	0.0765225	0.381797
CHRNA2	2.411521667	16.41214167	2.766748	0.0765225	0.381797
KLHDC8A	3.710156667	1.416797333	−1.38885	0.0765225	0.381797
SDHAF4	6.697633333	2.096109333	−1.67594	0.0765225	0.381797
DDX28	5.240941667	1.867753333	−1.48852	0.0765225	0.381797
FLG	5.70593	1.022835667	−2.47989	0.0765225	0.381797
GLYCTK	3.64294	1.456712333	−1.32239	0.0765225	0.381797
MTERF2	8.744611	1.822082	−2.26281	0.0765225	0.381797
DDIAS	7.581300667	1.137013667	−2.7372	0.0765225	0.381797
NIPAL2	7.583966667	1.822082	−2.05736	0.0765225	0.381797
QTRT1	6.379764	1.548054667	−2.04305	0.0765225	0.381797
SMYD5	9.282532667	1.822082	−2.34893	0.0765225	0.381797
AN-P2RY	4.527150333	1.844917667	−1.29505	0.0765225	0.381797
SEC61A2	4.182814667	1.388205333	−1.59125	0.0765225	0.381797
ARL6	7.930565	1.039818	−2.93109	0.0765225	0.381797
FAM179A	5.037166667	1.274027333	−1.98322	0.0765225	0.381797
SLC46A3	10.185989	1.091342333	−3.22241	0.0765225	0.381797
CAPN3	5.298772667	1.09807	−2.27069	0.0765225	0.381797
ITIH5	4.453339333	1.137013667	−1.96964	0.0765225	0.381797
FCN2	5.770094	1.525219	−1.91958	0.0765225	0.381797
ASB9	2.964853667	1.159849333	−1.35402	0.0765225	0.381797
ZNF90	4.634495667	1.068507	−2.11682	0.0765225	0.381797
MPP3	3.775197667	1.024517333	−1.88161	0.0765225	0.381797
JMJD7	3.819834333	1.068507	−1.83791	0.0765225	0.381797
PPIC	7.103771	1.182685	−2.58652	0.0765225	0.381797
PAK3	4.371605	1.639397333	−1.415	0.0765225	0.381797
MAP9	6.329609667	1.416797333	−2.15948	0.0765225	0.381797
NR2C2AP	3.038,413	1.388205333	−1.1301	0.0765225	0.381797
PIGV	4.787995	1.479548	−1.69427	0.0765225	0.381797
TNFAIP8L3	4.463735333	1.525219	−1.54924	0.0765225	0.381797

**Table:** 

HSD11B1L	4.341861	1.159849333	−1.90438	0.0765225	0.381797
ACPP	2.291559667	1.137013667	−1.01108	0.0765225	0.381797
RYR1	5.863586	1.296863	−2.17676	0.0765225	0.381797
ACKR4	6.268366	1.182685	−2.40602	0.0765225	0.381797
ZNF674	5.265564667	1.119454333	−2.23379	0.0765225	0.381797
SLC16A14	2.638718	1.228356	−1.10311	0.0765225	0.381797
NR6A1	1.286395667	5.917175	2.201574	0.0765225	0.381797
ABCB9	1.225025333	2.864761	1.225603	0.0765225	0.381797
GALNT18	1.204568333	3.584699	1.573336	0.0765225	0.381797
PRRT2	1.122741	7.446128	2.729465	0.0765225	0.381797
KHK	1.771375333	5.577208333	1.654673	0.0765225	0.381797
VSIG2	1.427099333	11.46590967	3.006193	0.0765225	0.381797
DYTN	2.382988333	13.68237767	2.521475	0.0765225	0.381797
KIAA1549	1.041696	5.577024333	2.420561	0.0765225	0.381797
NT5DC3	1.583743333	6.695642	2.079884	0.0765225	0.381797
GRB14	1.041696	19.04747167	4.192593	0.0765225	0.381797
SPHAR	1.833860667	8.911786667	2.280831	0.0765225	0.381797
CLDN12	1.437807667	4.590490667	1.674778	0.0765225	0.381797
DGKI	1.416959667	6.646423	2.229779	0.0765225	0.381797
TAF6L	1.142366333	4.517017333	1.983345	0.0765225	0.381797
LIPC	2.444002333	9.215349	1.914793	0.0765225	0.381797
PPP1R13L	1.166784	11.21800867	3.265207	0.0765225	0.381797
PLXNB3	3.989695	15.27107967	1.936452	0.0765225	0.381797

**Table:** Significant DEGs between T0 and T2

GENE	Mean(T0)	Mean(T2)	logFC	pValue	FDR
CCHCR1	7.595336	1	−2.92511	0.0636026	0.412752
CBR3	11.61466033	1	−3.53788	0.0636026	0.412752
CCDC40	5.298982333	1	−2.40572	0.0636026	0.412752
CLEC4C	4.453637333	1	−2.15498	0.0636026	0.412752
IL23A	6.11104	1	−2.61142	0.0636026	0.412752
WT1	5.004191667	1	−2.32314	0.0636026	0.412752
OC4-APO	3.817754	1	−1.93272	0.0636026	0.412752
APOC2	3.817754	1	−1.93272	0.0636026	0.412752
SMOC2	5.391121667	1	−2.43059	0.0636026	0.412752
APOC3	6.555501	1	−2.71271	0.0636026	0.412752
DTX3	4.302150667	1	−2.10506	0.0636026	0.412752
DHRS11	4.471552	1	−2.16078	0.0636026	0.412752
CCL20	3.247779667	1	−1.69945	0.0636026	0.412752
NLRP7	5.482675333	1	−2.45488	0.0636026	0.412752
GPA33	2.577333333	1	−1.36588	0.0636026	0.412752
ZBED6CL	1	2.731812667	1.449859	0.0636026	0.412752
PRRT3	1	2.731313667	1.449595	0.0636026	0.412752
SAA1	1	5.330368667	2.414235	0.0636026	0.412752
TCTEX1D1	1	5.456496	2.447975	0.0636026	0.412752
CEBPE	1	8.148108	3.026465	0.0636026	0.412752
DIRAS1	1	4.648991667	2.216918	0.0636026	0.412752
DEPDC4	1	5.40525	2.434361	0.0636026	0.412752
VNN3	1	4.804029667	2.264245	0.0636026	0.412752
WFDC1	1	11.938668	3.57757	0.0636026	0.412752
ADGRG3	1	5.034594	2.331875	0.0636026	0.412752
ILDR2	1	9.576249333	3.259461	0.0636026	0.412752
IFI27L1	1	5.030390667	2.33067	0.0636026	0.412752
OPLAH	1	4.975158667	2.314743	0.0636026	0.412752
TLR5	1	4.745244333	2.246482	0.0636026	0.412752
RETN	1	7.145587	2.837053	0.0636026	0.412752
MAK	1	5.000173	2.321978	0.0636026	0.412752
GCKR	1	6.585539	2.719302	0.0636026	0.412752
FBXO40	1	4.339207	2.117431	0.0636026	0.412752
MMP2	14.33027367	1.45041	−3.30453	0.0765225	0.412752
KCNH8	6.572459333	1.097483333	−2.58223	0.0765225	0.412752
PGAP2	7.833870333	1.311946667	−2.57802	0.0765225	0.412752
ZSWIM3	6.354159333	1.406925667	−2.17516	0.0765225	0.412752
KATNAL2	7.194896	1.194966667	−2.59	0.0765225	0.412752
GSTZ1	7.736256333	1.17547	−2.7184	0.0765225	0.412752
ARHGAP22	5.822747333	1.11698	−2.3821	0.0765225	0.412752
NEK3	10.27452633	1.857853667	−2.46736	0.0765225	0.412752
CYP2E1	9.410710667	1.214463333	−2.95398	0.0765225	0.412752
MMP23B	5.119569333	1.429937	−1.84007	0.0765225	0.412752
ABCA9	9.573765	1.757507667	−2.44556	0.0765225	0.412752
SCARB1	9.162249333	2.033324	−2.17186	0.0765225	0.412752
KIF5C	9.273256333	1.038993333	−3.15789	0.0765225	0.412752
SFT2D3	7.445747667	1.584900333	−2.23202	0.0765225	0.412752
C2orf81	6.706935333	1.389933667	−2.27064	0.0765225	0.412752
GZMK	7.516827	1.11698	−2.75052	0.0765225	0.412752
FGFR3	4.588419667	1.019377333	−2.17031	0.0765225	0.412752
ADTRP	4.90516	1.122839	−2.12715	0.0765225	0.412752
LRP5L	4.650881333	1.020473333	−2.18827	0.0765225	0.412752
CCR7	5.365534	1.245678333	−2.10679	0.0765225	0.412752
CSRP2	7.636079333	1.194966667	−2.67586	0.0765225	0.412752
ERP27	4.538457667	1.11698	−2.0226	0.0765225	0.412752
PLA2R1	3.766903667	1.143312333	−1.72016	0.0765225	0.412752
CLEC1A	5.842795333	1.506913667	−1.95506	0.0765225	0.412752

**Table:** 

RSPO3	11.824911	1.389933667	−3.08874	0.0765225	0.412752
C1orf56	3.535961667	1.019496667	−1.79425	0.0765225	0.412752
IDUA	3.500385	1.17547	−1.57428	0.0765225	0.412752
FOLR2	4.340185667	1.370437	−1.66312	0.0765225	0.412752
CHRNA2	2.411521667	25.994894	3.430213	0.0765225	0.412752
HSD17B3	2.288780667	6.393580667	1.482045	0.0765225	0.412752
C14orf80	4.038967333	1.584900333	−1.34959	0.0765225	0.412752
APOBEC3H	3.586465667	1.327571	−1.43377	0.0765225	0.412752
ADAMTSL3	3.572188	1.019496667	−1.80895	0.0765225	0.412752
C19orf44	5.222961667	1.682383667	−1.63436	0.0765225	0.412752
CERCAM	4.230867	1.573249	−1.42721	0.0765225	0.412752
FBLN1	8.063541	1.467920333	−2.45764	0.0765225	0.412752
FAM184A	7.253860333	1.584900333	−2.19436	0.0765225	0.412752
ZNF630	4.970839333	1.467920333	−1.75972	0.0765225	0.412752
C15orf65	2.889942	1.370437	−1.0764	0.0765225	0.412752
VPS9D1	1.838730333	5.310771667	1.530212	0.0765225	0.412752
ALG14	4.971241667	1.194966667	−2.05664	0.0765225	0.412752
ID4	7.315216667	1.081892667	−2.75734	0.0765225	0.412752
SPINT1	3.621315333	1.019377333	−1.82883	0.0765225	0.412752
MAP10	4.540429	1.584900333	−1.51844	0.0765225	0.412752
ABI3BP	7.148441	1.214463333	−2.55731	0.0765225	0.412752
RBKS	3.832568333	1.184258667	−1.69433	0.0765225	0.412752
DCAF4	1.490964	4.681461667	1.650714	0.0765225	0.412752
CIB2	2.768122667	1.05849	−1.3869	0.0765225	0.412752
GREB1L	4.893538333	1.467920333	−1.7371	0.0765225	0.412752
HSD11B1L	4.341861	1.467920333	−1.56454	0.0765225	0.412752
RYR1	5.863586	1.29245	−2.18167	0.0765225	0.412752
SNTG1	3.070525	1.389933667	−1.14347	0.0765225	0.412752
ACKR4	6.268366	1.429937	−2.13214	0.0765225	0.412752
ZNF835	4.583497333	1.23396	−1.89315	0.0765225	0.412752
TIFAB	7.848899333	1.102366	−2.83189	0.0765225	0.412752
NCBP2L	1.061370667	5.636411	2.408848	0.0765225	0.412752
FKBP10	1.040913667	2.451751333	1.235962	0.0765225	0.412752
DYTN	2.382988333	9.130906	1.937986	0.0765225	0.412752
MMP1	1.020848	4.238790333	2.053885	0.0765225	0.412752
SEC14L2	1.569465667	6.796782	2.114578	0.0765225	0.412752
MCEMP1	6.165867667	27.79493433	2.172446	0.0765225	0.412752
FICD	1.10424	5.152503	2.22222	0.0765225	0.412752
KIAA1549	1.041696	3.803068	1.868229	0.0765225	0.412752
ADORA2B	2.313422667	7.665795333	1.728407	0.0765225	0.412752
ARMC2	1.549127667	3.928262667	1.342435	0.0765225	0.412752
GRB14	1.041696	18.48755433	4.149548	0.0765225	0.412752
CEL	1.020848	10.00939533	3.293515	0.0765225	0.412752
SRPK3	1.325409	4.096300333	1.627884	0.0765225	0.412752
KCNMB1	1.711832333	12.59957767	2.879762	0.0765225	0.412752
DNAH2	1.750527333	8.483400667	2.276853	0.0765225	0.412752
DGKI	1.416959667	9.680691667	2.772311	0.0765225	0.412752
SDCBP2	1.062544	4.338208	2.029577	0.0765225	0.412752
WDSUB1	2.146639	8.531141667	1.990659	0.0765225	0.412752
WASF1	3.338877	15.118132	2.178845	0.0765225	0.412752
CRP	1.488113333	4.649315667	1.643534	0.0765225	0.412752
ZDHHC15	1.083392	3.372840667	1.638409	0.0765225	0.412752
ACCSL	1.610142	4.907580667	1.607824	0.0765225	0.412752
LRRC69	1.312719667	2.734634667	1.058789	0.0765225	0.412752
PLXNB3	3.989695	17.17698433	2.106126	0.0765225	0.412752
DGAT2	1.101690333	4.806447667	2.125252	0.0765225	0.412752
PIGV	4.787995	1.479548	−1.69427	0.0765225	0.381797
TNFAIP8L3	4.463735333	1.525219	−1.54924	0.0765225	0.381797

**Table:** 

HSD11B1L	4.341861	1.159849333	−1.90438	0.0765225	0.381797
ACPP	2.291559667	1.137013667	−1.01108	0.0765225	0.381797
RYR1	5.863586	1.296863	−2.17676	0.0765225	0.381797
ACKR4	6.268366	1.182685	−2.40602	0.0765225	0.381797
ZNF674	5.265564667	1.119454333	−2.23379	0.0765225	0.381797
SLC16A14	2.638718	1.228356	−1.10311	0.0765225	0.381797
NR6A1	1.286395667	5.917175	2.201574	0.0765225	0.381797
ABCB9	1.225025333	2.864761	1.225603	0.0765225	0.381797
GALNT18	1.204568333	3.584699	1.573336	0.0765225	0.381797
PRRT2	1.122741	7.446128	2.729465	0.0765225	0.381797
KHK	1.771375333	5.577208333	1.654673	0.0765225	0.381797
VSIG2	1.427099333	11.46590967	3.006193	0.0765225	0.381797
DYTN	2.382988333	13.68237767	2.521475	0.0765225	0.381797
KIAA1549	1.041696	5.577024333	2.420561	0.0765225	0.381797
NT5DC3	1.583743333	6.695642	2.079884	0.0765225	0.381797
GRB14	1.041696	19.04747167	4.192593	0.0765225	0.381797
SPHAR	1.833860667	8.911786667	2.280831	0.0765225	0.381797
CLDN12	1.437807667	4.590490667	1.674778	0.0765225	0.381797
DGKI	1.416959667	6.646423	2.229779	0.0765225	0.381797
TAF6L	1.142366333	4.517017333	1.983345	0.0765225	0.381797
LIPC	2.444002333	9.215349	1.914793	0.0765225	0.381797
PPP1R13L	1.166784	11.21800867	3.265207	0.0765225	0.381797
PLXNB3	3.989695	15.27107967	1.936452	0.0765225	0.381797

**Table:** 

NR6A1	1.286395667	3.191675333	1.310979	0.0765225	0.209152
DKK1	1.225025333	4.089077333	1.738964	0.0765225	0.209152
SCGB1C2	1.143198	5.987795333	2.38895	0.0765225	0.209152
GLIS3	1.122741	4.273733	1.928472	0.0765225	0.209152
NCBP2L	1.061370667	2.974784333	1.486856	0.0765225	0.209152
FKBP10	1.040913667	2.901915667	1.479155	0.0765225	0.209152
PPP1R14 C	1.062544	3.190217	1.586132	0.0765225	0.209152
GRTP1	2.972792	8.086871667	1.443763	0.0765225	0.209152
VSIG2	1.427099333	7.269909333	2.348,852	0.0765225	0.209152
DYTN	2.382988333	9.401901	1.980181	0.0765225	0.209152
MMP1	1.020848	7.486689333	2.87456	0.0765225	0.209152
SEC14L2	1.569465667	13.37922333	3.091649	0.0765225	0.209152
JSRP1	1.284733	3.192776333	1.313343	0.0765225	0.209152
TRIM16L	1.894874667	8.569771	2.177154	0.0765225	0.209152
FKBP14	1.166784	2.745736333	1.234656	0.0765225	0.209152
KIAA1549	1.041696	5.596469	2.425583	0.0765225	0.209152
TMEM17	1.081352333	2.765306333	1.354603	0.0765225	0.209152
SH3D21	1.081352333	2.318455333	1.100327	0.0765225	0.209152
ADORA2B	2.313422667	6.558939333	1.503434	0.0765225	0.209152
GRB14	1.041696	22.80897233	4.452595	0.0765225	0.209152
KIAA1211 L	1.10424	2.301469667	1.059502	0.0765225	0.209152
DYNC1I1	2.342312	21.485078	3.19733	0.0765225	0.209152
CEL	1.020848	4.892537667	2.260815	0.0765225	0.209152
SUCNR1	3.725300333	39.45302667	3.404707	0.0765225	0.209152
CRYM	1.162704333	6.564460333	2.497192	0.0765225	0.209152
SRPK3	1.325409	7.290892	2.459658	0.0765225	0.209152
KCNMB1	1.711832333	15.167389	3.147359	0.0765225	0.209152
SPHAR	1.833860667	5.496761	1.583698	0.0765225	0.209152
DNAH2	1.750527333	9.255194667	2.402474	0.0765225	0.209152
DGKI	1.416959667	4.966386333	1.809398	0.0765225	0.209152
WASF1	3.338877	33.65391	3.333339	0.0765225	0.209152
PRSS50	1.040676	11.52869267	3.469636	0.0765225	0.209152
HBE1	2.098255333	45.805628	4.448263	0.0765225	0.209152
PPP1R13L	1.166784	2.609403333	1.161182	0.0765225	0.209152
ACCSL	1.610142	12.29111	2.932355	0.0765225	0.209152
PLXNB3	3.989695	46.17292067	3.532697	0.0765225	0.209152
HOOK2	1.416959667	3.573092	1.334374	0.0765225	0.209152
DGAT2	1.101690333	2.642294333	1.262072	0.0765225	0.209152
FAM229A	1.061014333	3.219210333	1.601263	0.0765225	0.209152
ADORA2B	2.313422667	7.665795333	1.728407	0.0765225	0.412752
ARMC2	1.549127667	3.928262667	1.342435	0.0765225	0.412752
GRB14	1.041696	18.48755433	4.149548	0.0765225	0.412752
CEL	1.020848	10.00939533	3.293515	0.0765225	0.412752
SRPK3	1.325409	4.096300333	1.627884	0.0765225	0.412752
KCNMB1	1.711832333	12.59957767	2.879762	0.0765225	0.412752
DNAH2	1.750527333	8.483400667	2.276853	0.0765225	0.412752
DGKI	1.416959667	9.680691667	2.772311	0.0765225	0.412752
SDCBP2	1.062544	4.338208	2.029577	0.0765225	0.412752
WDSUB1	2.146639	8.531141667	1.990659	0.0765225	0.412752
WASF1	3.338877	15.118132	2.178845	0.0765225	0.412752
CRP	1.488113333	4.649315667	1.643534	0.0765225	0.412752
ZDHHC15	1.083392	3.372840667	1.638409	0.0765225	0.412752
ACCSL	1.610142	4.907580667	1.607824	0.0765225	0.412752
LRRC69	1.312719667	2.734634667	1.058789	0.0765225	0.412752
PLXNB3	3.989695	17.17698433	2.106126	0.0765225	0.412752
DGAT2	1.101690333	4.806447667	2.125252	0.0765225	0.412752
PIGV	4.787995	1.479548	−1.69427	0.0765225	0.381797
TNFAIP8L3	4.463735333	1.525219	−1.54924	0.0765225	0.381797

**Table:** Significant DEGs between T0 and T3

GENE	Mean(T0)	Mean(T3)	logFC	pValue	FDR
WT1	5.004191667	1	−2.32314	0.0636026	0.209152
CLEC1A	5.842795333	1	−2.54666	0.0636026	0.209152
APOC3	6.555501	1	−2.71271	0.0636026	0.209152
XG	2.106219667	1	−1.07466	0.0636026	0.209152
EYA1	9.177906	1	−3.19817	0.0636026	0.209152
PRRT3	1	3.799291667	1.92573	0.0636026	0.209152
P4HTM	1	2.007482	1.005387	0.0636026	0.209152
OR2B6	1	10.21555833	3.352696	0.0636026	0.209152
CAPN8	1	3.670159	1.875843	0.0636026	0.209152
GLDC	1	3.748804	1.90643	0.0636026	0.209152
TCTEX1D1	1	3.947338	1.98088	0.0636026	0.209152
RNF215	1	2.582703333	1.368882	0.0636026	0.209152
DIRAS1	1	4.19538	2.068801	0.0636026	0.209152
KCNQ4	1	2.161521333	1.112047	0.0636026	0.209152
DEPDC4	1	3.900750333	1.963752	0.0636026	0.209152
GRIK5	1	3.591186	1.84446	0.0636026	0.209152
GFAP	1	2.811727	1.491457	0.0636026	0.209152
SLC29A2	1	2.196367667	1.13512	0.0636026	0.209152
NKAIN2	1	4.292667667	2.101874	0.0636026	0.209152
HTR2A	1	4.016981333	2.006112	0.0636026	0.209152
WFDC1	1	3.885309667	1.95803	0.0636026	0.209152
ZMYND12	1	3.471236667	1.79545	0.0636026	0.209152
PGA3	1	2.335415	1.223679	0.0636026	0.209152
KBTBD12	1	2.296444667	1.199402	0.0636026	0.209152
ILDR2	1	10.93074	3.450319	0.0636026	0.209152
IFI27L1	1	2.527466	1.337692	0.0636026	0.209152
OPLAH	1	3.064273667	1.615545	0.0636026	0.209152
IGSF21	1	2.188809	1.130146	0.0636026	0.209152
KCNA2	1	3.154114333	1.657235	0.0636026	0.209152
TLR5	1	3.060803667	1.613911	0.0636026	0.209152
VIPR2	1	6.112661333	2.611801	0.0636026	0.209152
HIST1H1T	1	4.333762667	2.11562	0.0636026	0.209152
SLC4A11	1	3.405952333	1.768058	0.0636026	0.209152
MAK	1	2.680692333	1.422606	0.0636026	0.209152
CDHR1	1	25.677904	4.682456	0.0636026	0.209152
PPP1R32	1	4.505728667	2.17176	0.0636026	0.209152
GCKR	1	3.954844667	1.983621	0.0636026	0.209152
FBXO40	1	2.491368333	1.316938	0.0636026	0.209152
KCNH8	6.572459333	1.080832667	−2.60429	0.0765225	0.209152
DZIP1	7.629405	1.424371667	−2.42124	0.0765225	0.209152
ARHGAP22	5.822747333	1.086327667	−2.42224	0.0765225	0.209152
NKX2-3	4.342062	1.383955333	−1.64958	0.0765225	0.209152
PLA2R1	3.766903667	1.060624667	−1.82846	0.0765225	0.209152
SLC2A4	9.014120333	1.014388	−3.15158	0.0765225	0.209152
OC4-APO	3.817754	1.071939667	−1.8325	0.0765225	0.209152
APOC2	3.817754	1.071939667	−1.8325	0.0765225	0.209152
CRTAC1	6.897075667	1.201323	−2.52136	0.0765225	0.209152
CHRNA2	2.411521667	13.60272067	2.49588	0.0765225	0.209152
LAYN	6.237966	1.028776	−2.60015	0.0765225	0.209152
SMOC2	5.391121667	1.083884667	−2.31437	0.0765225	0.209152
FAM155A	4.772379	1.101041	−2.11584	0.0765225	0.209152
IFNG	3.556483333	1.028776	−1.78952	0.0765225	0.209152
VPS9D1	1.838730333	11.01438633	2.582607	0.0765225	0.209152
SLC35D3	1.572791333	4.819860333	1.615664	0.0765225	0.209152
LRFN4	3.209380667	1.115103333	−1.52512	0.0765225	0.209152
ACKR4	6.268366	1.101041	−2.50922	0.0765225	0.209152
CYS1	1.38868	5.606796333	2.013462	0.0765225	0.209152

**Table:** 

HSD11B1L	4.341861	1.159849333	−1.90438	0.0765225	0.381797
ACPP	2.291559667	1.137013667	−1.01108	0.0765225	0.381797
RYR1	5.863586	1.296863	−2.17676	0.0765225	0.381797
ACKR4	6.268366	1.182685	−2.40602	0.0765225	0.381797
ZNF674	5.265564667	1.119454333	−2.23379	0.0765225	0.381797
SLC16A14	2.638718	1.228356	−1.10311	0.0765225	0.381797
NR6A1	1.286395667	5.917175	2.201574	0.0765225	0.381797
ABCB9	1.225025333	2.864761	1.225603	0.0765225	0.381797
GALNT18	1.204568333	3.584699	1.573336	0.0765225	0.381797
PRRT2	1.122741	7.446128	2.729465	0.0765225	0.381797
KHK	1.771375333	5.577208333	1.654673	0.0765225	0.381797
VSIG2	1.427099333	11.46590967	3.006193	0.0765225	0.381797
DYTN	2.382988333	13.68237767	2.521475	0.0765225	0.381797
KIAA1549	1.041696	5.577024333	2.420561	0.0765225	0.381797
NT5DC3	1.583743333	6.695642	2.079884	0.0765225	0.381797
GRB14	1.041696	19.04747167	4.192593	0.0765225	0.381797
SPHAR	1.833860667	8.911786667	2.280831	0.0765225	0.381797
CLDN12	1.437807667	4.590490667	1.674778	0.0765225	0.381797
DGKI	1.416959667	6.646423	2.229779	0.0765225	0.381797
TAF6L	1.142366333	4.517017333	1.983345	0.0765225	0.381797
LIPC	2.444002333	9.215349	1.914793	0.0765225	0.381797
PPP1R13L	1.166784	11.21800867	3.265207	0.0765225	0.381797
PLXNB3	3.989695	15.27107967	1.936452	0.0765225	0.381797

In short, this report presented abundant exLRs in human plasma and the exLR dynamic changes. ExLRs originating from CD8 + T and CD4+ memory T cells were reduced during the perioperative period. Future studies will focus on the specific exLRs dynamically changed during the perioperative period and their origins to explore the impact on the occurrence and progression of CRC and the potential underlying mechanism.

## Conclusions

To the best of our knowledge, we investigated the effects of surgical stress on the expression profile and cellular sources of blood exLR by exLR sequencing of CRC patients at four time points before and after surgery. In addition, we also investigated the function of these changed exLRs during the perioperative period. These findings open an avenue for the investigation of EVs at different time points and lay foundation to find out exLRs involved in the postoperative metastasis and recurrence of tumors.

## Supplementary Material

Supplemental Material

## Highlights

- Eleven DEGs and three DELRs dynamically changed during perioperative period.
- The dynamically changed exLRs were related to various biofunctions.
- EVs originating from immune cells decreased after surgery.
