# Supplementary material for: Dynamic changes of plasma extracellular vesicle long RNAs during perioperative period of colorectal cancer
Source: Bioengineered. 2021 Jul 16;12(1):3699–710. doi: 10.1080/21655979.2021.1943281 (PMC8806447; doi:10.1080/21655979.2021.1943281)
Supplement: Supplemental Material [file KBIE_A_1943281_SM5043.zip › Supplementary materialsclean.docx]

**Supplementary materials**

**Table1** **Comprehensive mRNAs in EVs before and after surgery.** The details of differentially expressed genes (DEGs), including gene expression, logFC, pValue and FDR.

**Table2 Comprehensive lncRNAs in EVs before and after surgery.** The details of differentially-expressed lncRNAs (DELRs), including gene expression, logFC, pValue and FDR.

Fig S1. **Volcano plot of comprehensive mRNAs in EVs before and after surgery.** (A-C) Comparing of DEGs in extracellular vesicles were screened and identified using the “Limma” R package between samples before surgery and after extubation (Fig. A), or 1 day after surgery (Fig. B), or 3 days after surgery (Fig. C).

Fig S2. **Volcano plot of comprehensive lncRNAs in EVs before and after surgery.** (A-C) Comparing of lncRNAs in extracellular vesicles were screened and identified using the “Limma” R package between samples before surgery and after extubation (Fig. A), or 1 day after surgery (Fig. B), or 3 days after surgery (Fig. C).
