# Supplementary figures and images for: Dynamic changes of plasma extracellular vesicle long RNAs during perioperative period of colorectal cancer
Source: Bioengineered. 2021 Jul 16;12(1):3699–710. doi: 10.1080/21655979.2021.1943281 (PMC8806447; doi:10.1080/21655979.2021.1943281)

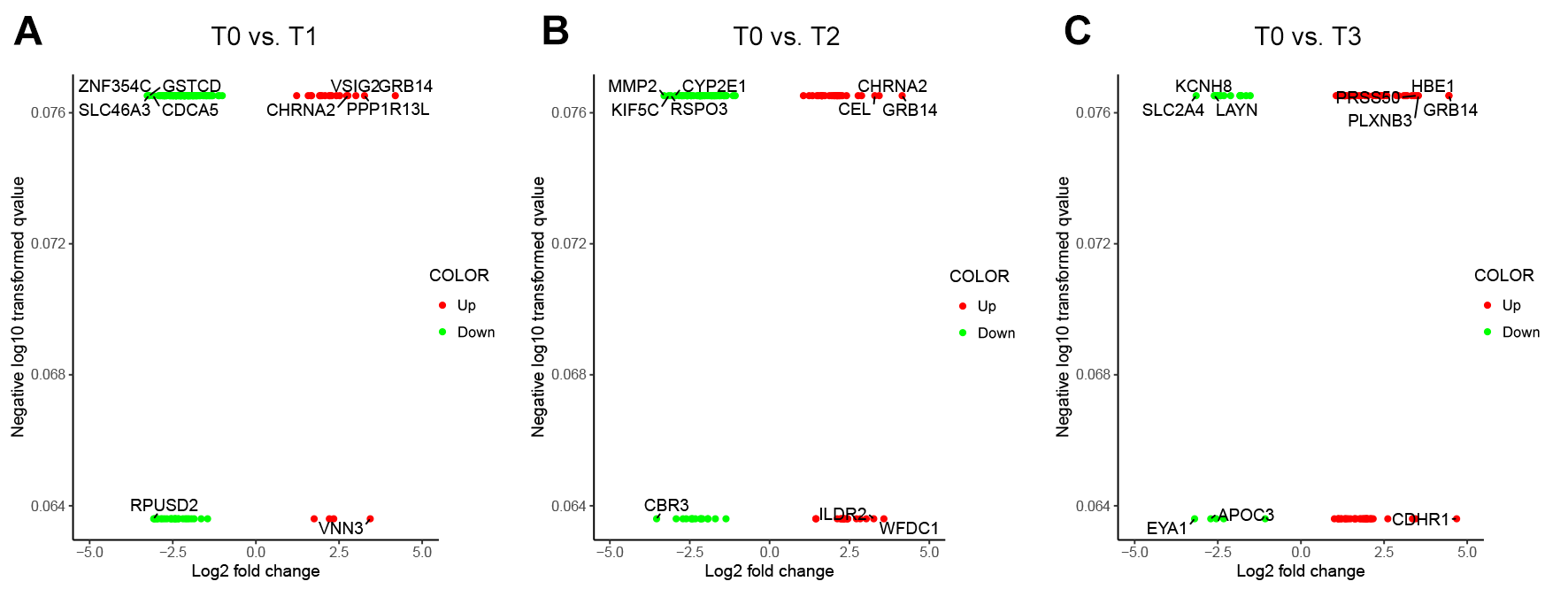

Supplement: Supplemental Material [file KBIE_A_1943281_SM5043.zip › S1.tif]

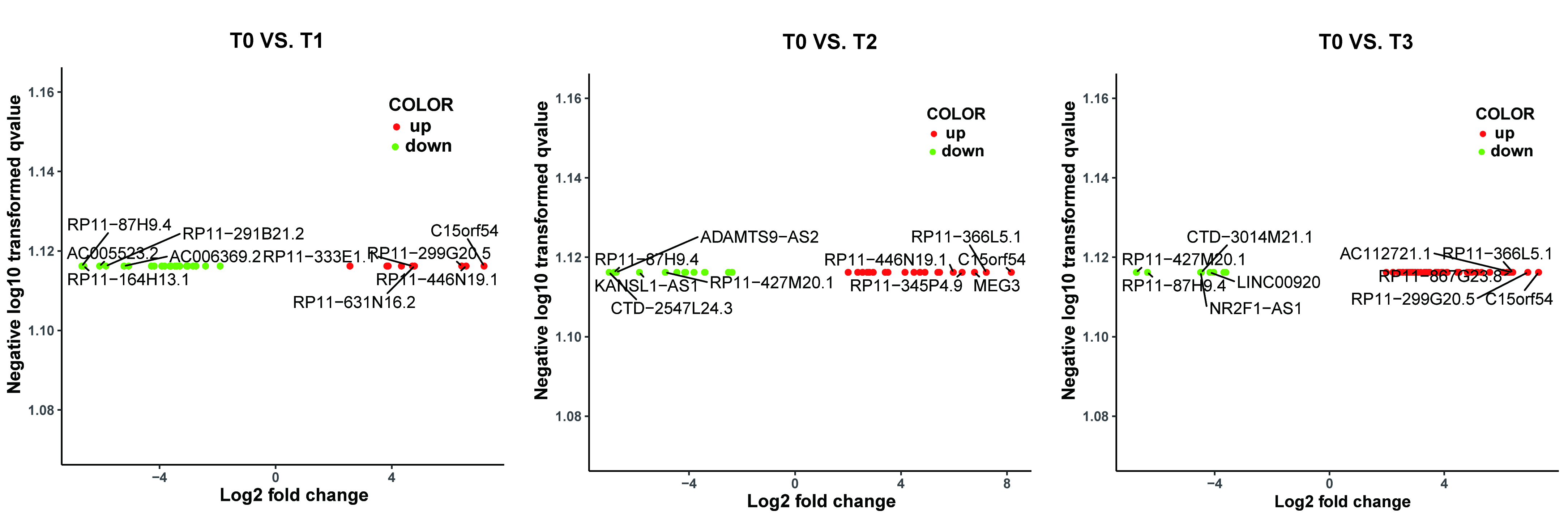

Supplement: Supplemental Material [file KBIE_A_1943281_SM5043.zip › S2.tif]
